# Modification of Growth and Physiological Response of Coastal Dune Species *Anthyllis maritima* to Sand Burial by Rhizobial Symbiosis and Salinity

**DOI:** 10.3390/plants10122584

**Published:** 2021-11-25

**Authors:** Laura Gaile, Una Andersone-Ozola, Ineta Samsone, Didzis Elferts, Gederts Ievinsh

**Affiliations:** 1 Department of Plant Physiology, Faculty of Biology, University of Latvia, 1 Jelgavas Str., LV-1004 Riga, Latvia; laura_lg@inbox.lv (L.G.); una.andersone-ozola@lu.lv (U.A.-O.); ineta.samsone@inbox.lv (I.S.); 2 Department of Botany and Ecology, Faculty of Biology, University of Latvia, 1 Jelgavas Str., LV-1004 Riga, Latvia; didzis.elferts@lu.lv

**Keywords:** *Anthyllis maritima*, coastal species, rhizobial symbiosis, salinity, sand burial, wild legumes

## Abstract

The aim of the present study was to establish an experimental system in controlled conditions to study the physiological effect of abiotic/biotic interaction using a rare wild leguminous plant species from coastal sand dunes, *Anthyllis maritima*. The particular hypothesis tested was that there is an interaction between sand burial, rhizobial symbiosis and salt treatment at the level of physiological responses. Experiment in controlled conditions included 18 treatment combinations of experimental factors, with two intensities of sand burial, rhizobial inoculation and two types of NaCl treatment (soil irrigation and foliar spray). Shoot biomass was significantly affected both by burial and by inoculation, and by interaction between burial and NaCl in the case of shoot dry mass. For plants sprayed with NaCl, burial had a strong significant positive effect on shoot growth irrespective of inoculation. General effect of inoculation with rhizobia on shoot growth of plants without NaCl treatment was negative except for the plants buried 2 cm with sand, where significant stimulation of shoot dry mass by inoculant was found. The positive effect of burial on shoot growth was mainly associated with an increase in leaf petiole height and number of leaves. Performance index significantly increased in buried plants in all treatment combinations, and leaf chlorophyll concentration increased in buried plants independently on burial depth, and only in plants not treated with NaCl. Inoculation led to significant increase of leaf peroxidase activity in all treatment combinations except NaCl-irrigated plants buried for 2 cm by sand. Sand burial stimulated peroxidase activity, mostly in non-inoculated plants, as inoculation itself led to increased enzyme activity. In conclusion, strong interaction between sand burial and NaCl treatment was evident, as the latter significantly affected the effect of burial on growth and physiological indices. Moreover, rhizobial symbiosis had a significant effect on physiological processes through interaction with both sand burial and NaCl treatment, but the effect was rather controversial; it was positive for photosynthesis-related parameters but negative for growth and tissue integrity indices.

## 1. Introduction

Environmental factors in coastal habitats form specific conditions requiring particular morphological and physiological adaptations of coastal plants, which is one of the reasons why many rare plant species are found there [1,2]. In coastal areas where sand dunes are formed, plant burial by sand represents the main specific environmental factor affecting growth and physiological performance of plants [3], leading to specific vegetation zonation patterns [4]. However, the physiological mechanisms of how dune plants respond and adapt to sand burial are far from clear [5]. In addition to wind-driven sand movement, seawater-related effects are often considered in respect to sand dune plants due to a proximity to the sea [6]. In contrast to coastal marshes where seawater flooding directly affects substrate salinity, sand dune plants are subjected mostly to saltwater spray [7].

Shortage of plant-available nitrogen in soil is one of the plant growth-limiting factors in coastal dunes [8] pointing to the possible advantages of symbiosis with N_2_-fixing bacteria [9]. The importance of symbiotic N_2_-fixing relationships for wild legume species has been studies mostly in respect to mineral nutrition, but information is available also on how rhizobial symbiosis affects responses of leguminous plants to unfavourable environmental conditions, as in arid [10] or saline [11,12] regions. Several experiments have suggested different responses of symbiotic bacteria and host plants to salinity [13]. Recently, it was established that rhizobial symbiosis significantly affects the outcome of coexistence of *Trifolium fragiferum* and *Trifolium repens* on the background of increased soil salinity [14]. To our knowledge, there is no data available on the effects of rhizobial symbiosis based on studies with wild plants from coastal dune habitats. 

It can be argued that interactions between different factors affect both distribution and physiological responses of coastal plants. Usually, it is difficult to reveal mechanisms of these interactions in field conditions, due to covariation of many factors caused by prevalent environmental gradients in a coastal area [3]. However, experimental evidence from multi-factor experiments in controlled conditions providing cause and effect relationship is scarce. One example of this type of study on responses of coastal plants to the concomitant influence of sand deposition and saltwater spray include experiments with an annual dune grass species *Triplasis purpurea* [15]. 

*Anthyllis maritima* Schweigg. (syn. *Anthyllis vulneraria* subsp. *maritima* (Hagen) Corb.) is a perennial legume species endemic to the Baltic Sea region [16]. Genus *Anthyllis* has complex and disputable intrageneric taxonomy [17] and certain species have been studied mostly as medicinal plants [18]. Recently, some taxa of the genus have gained attention for a potential use in phytoremediation [19]. While species similar to *Anthyllis vulneraria* are characteristic plants of dry calcareous grasslands, *A. maritima* is almost exclusively associated with coastal or inland sand dunes [16]. It is an important floristic component of both semi-stabilized white as well as grey coastal dunes; however, how environmental factors of coastal sand dunes affect growth and physiological performance of *A. maritima* has not been experimentally assessed.

In the case of studies with wild plant species possessing putative physiological adaptations to prevailing environmental factors, it is important to use suitable plant traits with a high level of informative utility for quantitative predictions of plant performance and reproduction success. Photosynthesis-related parameters have been widely used in ecophysiological studies of wild plants. Thus, photosynthetic performance of growing intact plants can be characterized nondestructively by an analysis of photochemical activity through measurement of chlorophyll *a* fluorescence [20] and, indirectly, by analysis of leaf chlorophyll concentration [21,22]. In addition, peroxidase and polyphenol oxidase are two enzymes involved in oxidative metabolism. They are frequently used to characterize general plant responses to unfavorable environmental conditions [23,24]. 

The aim of the present study was to establish an experimental system in controlled conditions to study physiological effect of abiotic/biotic interaction using a rare wild leguminous plant species from coastal sand dunes, *A. maritima*. The particular hypothesis tested was that there is an interaction between sand burial, nitrogen-fixing rhizobial symbiosis and salt treatment at the level of physiological responses. 

## 2. Materials and Methods

### 2.1. Plant Material, Growth Conditions and Treatments

Seeds of *A. maritima* were collected at the end of August 2016 from plants growing at coastal sand dunes near Užava, Latvia. Experiments were performed during winter season in partially controlled conditions. Seeds were dried at room temperature for three weeks and stored at 4 °C. Before germination, seeds were surface sterilized with 50% Ace (Procter & Gamble, Warszawa, Poland) for 7 min followed by washing in sterile deionized water (10 × 2 min), and were scarified using a scalpel. Scarified seeds were imbibed in a sterile deionized water for 4 h and sown in sterile plastic tissue culture containers with 1 cm autoclaved commercial garden soil (Biolan, Eura, Finland) mixed with sterile deionized water. Containers were stored in a growth cabinet with 16 h photoperiod (100 µmol m^–2^ s^–1^) at 23 °C. Seedlings were transplanted to sterilized 200 mL plastic containers filled with autoclaved commercial garden soil (Biolan, Eura, Finland) mixed with sterile deionized water after the appearance of the first two true leaves. Containers were placed in sterilized 48 L plastic boxes closed with lids in an experimental automated greenhouse (HortiMax, Maasdijk, Netherlands) with supplemented light from Master SON-TPIA Green Power CG T 400 W (Philips, Amsterdam, Netherlands) and Powerstar HQI-BT 400 W/D PRO (Osram, Munich, Germany) lamps (380 µmol m^–2^ s^–1^ at the plant level), 16 h photoperiod, day/night temperature 23/16 °C, relative air humidity 60 to 70%. After two weeks, 108 individual plants were transplanted in 1.2 L plastic containers filled with autoclaved quartz sand (Saulkalne-S, Saulkalne, Latvia), up to half the height followed by 2 cm commercial garden soil (Biolan, Eura, Finland). 

Half of the plants were inoculated with rhizobia isolated from roots of wild-grown *A. maritima* plants found at the same site where the seeds were collected. Plant roots were surface-sterilized in 50% commercial bleach (ACE, Procter & Gamble, Warszawa, Poland) for 15 min and rinsed thoroughly with sterile deionized water four times. Bacterial cells were isolated from detached nodules using the standard protocol on yeast-mannitol agar [25]. Bacterial suspension (about 10^9^ bacterial cells per mL) was applied 6 mL per container, evenly distributing 1 mL of suspension in six points over the surface of the substrate. All procedures were performed in a laminar box using sterilized instruments and material. 

After transplanting and bacterial inoculation, plants were cultivated in a greenhouse in conditions as described above. Individual containers were randomly placed in a greenhouse and were repositioned twice a week. Plants were irrigated with deionized water twice a week, not allowing a decrease of substrate moisture lower than 60% water holding capacity. Fertilization was performed weekly with a Kristalon Green Label fertilizer (NPK 18-18-18 with micronutrients; Yara International, Oslo, Norway) solubilized in deionized water (150 g L^–1^), with 5 mL of stock solution per L, 120 mL of the final fertilizer per container. Nitrogen was present in the fertilizer, both in a form of nitrate-N (9.8%) and ammonia-N (8.2%). Individual watering systems of containers were used to decrease possible contamination with rhizobial bacteria in the early stages of the experiment, where each container had a plastic plate under it for the accumulation of excessive water. Substrate water content was monitored with an HH2 moisture meter equipped with a WET-2 sensor (Delta-T Devices, Burwell, UK). 

One week after inoculation, both inoculated (+i) and non-inoculated (−i) plants were divided for further treatment with NaCl in a form of soil irrigation or foliage spray. For treatment, an individual container was irrigated with 100 mL 50 mM NaCl (NaI) or foliage of a plant was sprayed with 25 mL 100 mM NaCl (NaS). Respective untreated plants were irrigated and sprayed with an appropriate volume of deionized water. 

**Figure 1:**
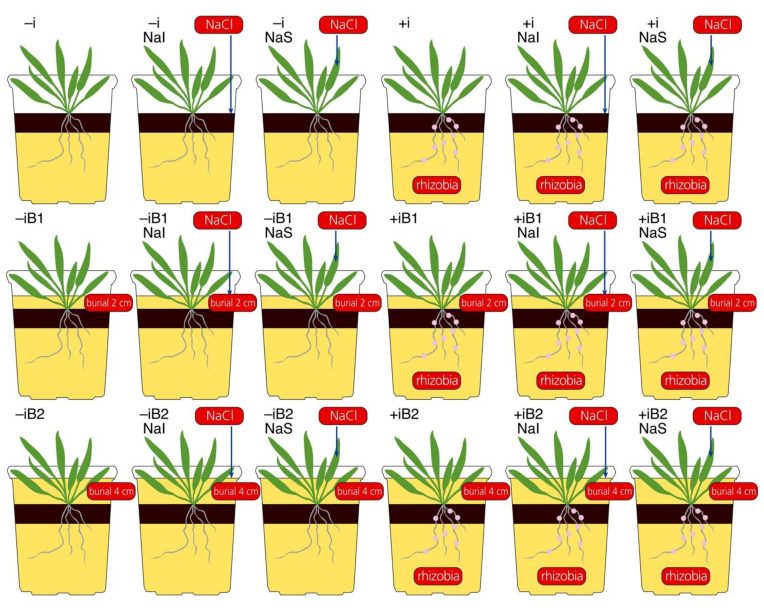
Combination of factors resulting in 18 treatments used in a study with *Anthyllis maritima* in sterile substrate. –i, no rhizobial inoculant; +i, with rhizobial inoculant; NaI, irrigated with 25 mM NaCl; NaS, sprayed with 100 mM NaCl; B1, buried with sand by 2 cm; B2, buried with sand by 4 cm.

In the next day, plants from all treatment combinations were further divided for sand burial treatment: without burial, buried with autoclaved quartz sand for 2 cm (B1), and buried for 4 cm (B2). Plant height at the time of burial was 5.4 ± 0.4 cm. Consequently, relative burial depth for 2 and 4 cm burial was 37% and 74% of plant height, respectively. As a result, 18 treatment combinations were established, each with six individual plants (Figure 1). One week after burial, respective plants were repeatedly treated with NaCl using the same procedure as described above. This week was designated as week 1, as analysis of physiological parameters had also started on the same day. 

### 2.2. Analysis of Physiological Parameters

Measurement of leaf chlorophyll concentration and chlorophyll *a* fluorescence analysis in leaves was performed once a week for six weeks. Four plants per treatment were randomly selected each week for the analysis. For every individual, three fully grown non-senescent leaves were selected. Chlorophyll concentration was measured by a chlorophyll meter CCM-300 (Opti-Sciences, Hudson, NH, USA). Chlorophyll *a* fluorescence was measured in leaves dark-adapted for at least 20 min by Handy PEA fluorometer (Hansatech Instruments, King’s Lynn, UK). Chlorophyll *a* fluorescence parameter performance index (total) was used for the characterization of photochemical activity, which combines three function-related (trapping of absorbed exciton, electron transport between the photosystems, reduction of end-electron acceptors) and one structure-related (antenna chlorophyll per reaction center chlorophyll) parameter [26].

### 2.3. Analysis of Ion Concentration

On week 2, 4 and 6, one fully grown leaf per plant was collected for measurement of tissue ion concentration [27]. Plant material from each two plants was pooled, and three replicates per treatment were analyzed. Leaves were dried at 60 °C in an oven until a constant mass, crushed by hand to small pieces, and a sample of 0.2 g was randomly taken from the total amount of leaf material. Tissues were ground with mortar and pestle to a fine powder and 10 mL of deionized water was added. The homogenate was stirred with pestle for 1 min. After filtration through nylon mesh cloth (No. 80), homogenate was used for measurement of ion concentration by LAQUAtwin compact meters B-722 (Na^+^) and B-731 (K^+^), and electrical conductivity (EC) by LAQUAtwin conductivity meter B-771 (Horiba, Kyoto, Japan).

### 2.4. Plant Harvest

The experiment was terminated on week 7. Plant shoots were cut and morphological parameters (fresh mass, number of shoots, number of leaves, leaf blade height, leaf petiole height) were measured for all individual plants. Fresh leaf samples (one leaf per plant, three replicates per treatment) were collected for biochemical analysis, quickly frozen in liquid nitrogen and stored at −20 °C. Plant roots were carefully freed from substrate, washed and their fresh mass was measured. Plant tissues were dried to constant mass in an oven at 60 °C and dry mass was measured. Shoot and root water content was estimated as a mass of water per dry mass of tissues.

### 2.5. Measurement of Biochemical Parameters

All biochemical measurements were performed in three replicates per treatment. 

Malondialdehyde (MDA) content was estimated by measurement of concentration of thiobarbituric acid-reactive substances (TBARS) according to Aref et al. [28]. Briefly, frozen plant material was ground with mortar and pestle to a fine powder and extracted with 0.1% trichloroacetic acid (10 mL g^–1^ FM), centrifuged at 12,000× *g* at 4 °C. To 1 mL of supernatant 4 mL of 0.5% thiobarbituric acid in 20% trichloroacetic acid was added and heated for 30 min at 90 °C. After rapid cooling in ice bath, the mixture was centrifuged at 10,000× *g* for 5 min at room temperature and absorbance was measured at 532 and 600 nm. 

Relative electrolyte leakage was measured according to Luo et al. [29]. Leaf discs (15 per analysis, 0.5 cm^2^ each) were prepared from fresh leaves, rinsed with deionized water three times and immersed in tubes with 10 mL deionized water for 22 h at room temperature. After that, electrical conductivity of the solution was measured using LAQUAtwin compact conductivity meter B-771 (Horiba, Kyoto, Japan). Tubes were incubated in a water bath at 80 °C for 2 h, cooled to room temperature and final conductivity was measured. 

Enzyme activities were measured using a frozen plant material according to Andersone and Ievinsh [30]. Briefly, for preparation of extracts, plant tissue was frozen in liquid nitrogen, ground with mortar and pestle to a fine powder and extracted with 25 mM HEPES/KOH buffer (pH 7.2) containing 1 mM EDTA, 3% polyvinylpolypirrolidone, 0.8% Triton X-100 (5 mL of buffer per g FM) for 15 min. After centrifugation at 15,000× *g* at 4 °C for 20 min, supernatant was used for measurement of enzyme activity. Peroxidase activity was measured using guaiacol and H_2_O_2_ as substrates, and polyphenol oxidase activity using catechol.

### 2.6. Data Analysis

For all dependent variables, homogeneity of variance was tested using a Levene test. If violation was observed then variables were log-transformed. Analysis of variance (ANOVA) was used to test main effects, as well as all two- and three-way interactions of factors: species, NaCl, and inoculant, if there were enough observations in each combination. A separate ANOVA model was developed for each dependent variable. For significant variables, Tukey HSD tests were used as post hoc tests to determine significant differences between factor levels. All analyses were performed using software R 4.1.1 [31].

## 3. Results

### 3.1. Morphological Parameters

**Figure 2:**
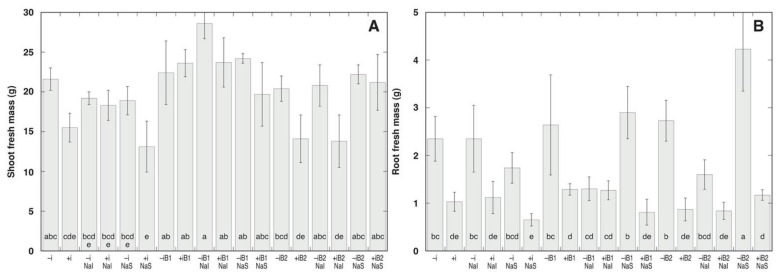
Fresh mass of shoots (**A**) and fresh mass of roots (**B**) of *Anthyllis maritima* plants after seven weeks of cultivation in the respective combination of conditions. –i, no rhizobial inoculant; +i, with rhizobial inoculant; NaI, irrigated with 25 mM NaCl; NaS, sprayed with 100 mM NaCl; B1, buried with sand by 2 cm; B2, buried with sand by 4 cm. Different letters indicate significant differences (*p* < 0.05) between treatments.

Both fresh and dry mass of shoots of *A. maritima* plants varied significantly between the treatments (Figure 2, Table 1). The weakest shoot growth was seen in non-buried inoculated plants sprayed with NaCl (+iNaS) and inoculated plants buried with 4 cm of sand and irrigated with NaCl (+iB2NaI). The most pronounced shoot growth was in the case of non-inoculated plants buried with 2 cm of sand and irrigated with NaCl (–iB1NaI). Root growth was also a highly variable parameter, with the lowest values in non-buried inoculated plants sprayed with NaCl (+iNaS). The highest root growth, 6.5 times the lowest value for root fresh mass, was evident in non-inoculated plants buried with 4 cm of sand and sprayed with NaCl (–iB2NaS). 

According to the ANOVA results, shoot fresh and dry mass was significantly affected both by burial and inoculation, and by interaction between burial and NaCl in the case of shoot dry mass (Table 2). Moderate burial (2 cm) had positive effect on shoot growth of plants without NaCl treatment only without inoculation, but 4 cm sand burial had no significant effect. When plants were irrigated with NaCl, 2 cm, burial had stimulative effect on shoot growth irrespective of bacterial inoculation, but 4 cm burial had a negative effect only in inoculated plants. For plants sprayed with NaCl, burial had strong positive effects on shoot growth irrespective of inoculation. The general effect of inoculation with rhizobia on the shoot growth of plants without NaCl treatment was negative, except for the plants buried 2 cm with sand, where stimulation of shoot dry mass by inoculant was found. For plants irrigated with NaCl, inoculant had a negative effect on shoot growth only in the case of sand burial, and this effect was more severe with increased burial depth. In contrast, when plants were sprayed with NaCl, the negative effect of inoculation decreased with sand burial and there was no significant effect of inoculation for 4 cm sand buried plants. NaCl treatment had a significant effect on shoot growth only in some treatment combinations. Thus, NaCl irrigation stimulated shoot growth of non-inoculated plants buried for 2 cm by sand. This effect was less pronounced in the case of NaCl spray.

According to ANOVA results, root mass was significantly affected only by inoculant (Table 2). Inoculation led to the significant inhibition of root growth in all treatment combinations, but this effect was partially modified by burial and NaCl treatment in a form of substrate irrigation. Thus, plants buried by 2 cm of sand and irrigated with NaCl showed no negative root growth response to bacterial inoculation. Detailed analysis of relative effects of different factors revealed that sand burial and NaCl treatment also affected root growth in some treatment combinations. Burial had a mostly non-significant effect in plants receiving no NaCl treatment, except in inoculated plants at 2 cm burial, where it was significantly negative. However, burial effect on root growth for NaCl-sprayed plants was significantly positive, especially for fresh mass. NaCl irrigation had no significant effect on root growth of bacteria-inoculated plants, but the effect was significantly negative in non-inoculated sand-buried plants. In contrast, NaCl spray had a significantly negative effect on root fresh mass of non-inoculated plants without burial; however, the effect was positive in the case of sand burial, while significant only in the case of 4 cm sand. In inoculated plants, NaCl spray had a negative effect on root growth without burial or with 2 cm burial, but a positive effect with 4 cm burial. 

Rhizobial inoculant tended to reduce shoot water content of unburied plants and plants buried for 2 cm, except unburied plants irrigated with NaCl and 2-cm-buried plants sprayed with NaCl (Table 1). Burial by 2 cm resulted in higher shoot water content in all treatment combinations; however, in 4-cm-buried plants this effect was evident only without rhizobial inoculation. In the roots of unburied plants, tissue water content increased by irrigation with NaCl (Table 1). 

Shoot morphological characteristics were analyzed in detail in order to understand which plant parts were affected by the experimental factors. Positive effect of burial on shoot growth was mainly associated with an increase in leaf petiole height and number of leaves (Table 1). Petiole height was significantly higher in sand-buried plants, with the highest value in non-inoculated plants buried at 4 cm. According to the results of ANOVA, leaf height was not significantly affected by any of the experimental factors, but petiole height was significantly affected only by burial (Table 3). Number of leaves was significantly affected by burial, inoculant and interaction between burial and NaCl, as in the case of shoot DM; however, the number of shoots was affected by burial, NaCl, an interaction between burial and NaCl, as well as by three-way interaction between burial, inoculant and NaCl. Inoculation negatively affected petiole height only in 4 cm buried plants without NaCl treatment. The positive effect of inoculation on number of leaves and number of shoots was seen in the case of 2 cm burial without NaCl treatment; however, in plants irrigated with NaCl, the effect was negative in respect to the number of leaves of buried plants and positive for the number of shoots of non-buried plants. The effect of burial on morphological characteristics was somehow controversial, as in contrast to positive influence on petiole height, the effect of burial on the number of leaves changed with burial depth and the presence of inoculant. Burial at depth of 4 cm negatively affected the number of leaves in plants without NaCl, or irrigated by NaCl, but NaCl spray eliminated this effect. In general, NaCl treatment itself was neutral or had a positive effect on shoot morphological characteristics. NaCl irrigation stimulated petiole height, number of leaves and number of shoots mostly in non-inoculated plants in burial conditions, but NaCl spray was relatively less effective.

### 3.2. Physiological Parameters

**Figure 3:**
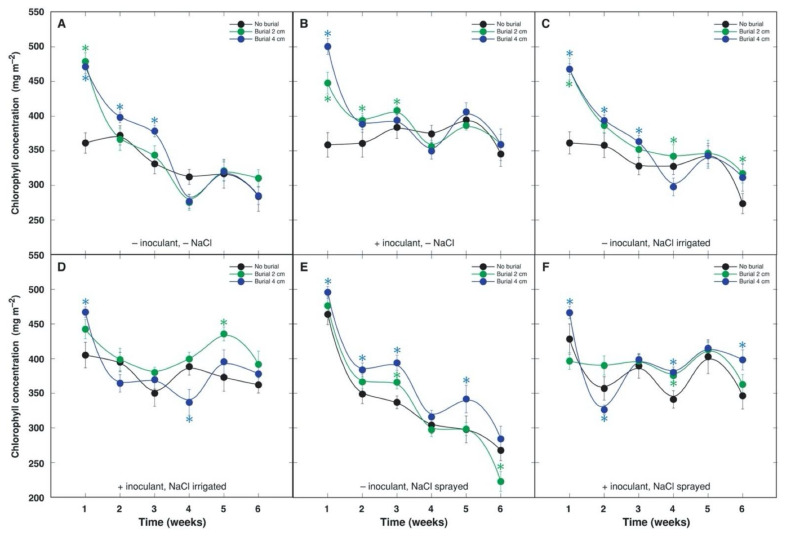
Sand burial effect on time course of chlorophyll concentration in leaves of *Anthyllis maritima* without rhizobial inoculant and without NaCl treatment (**A**), with rhizobial inoculant and without NaCl treatment (**B**), without rhizobial inoculant and with NaCl treatment in a form of substrate irrigation (**C**), with rhizobial inoculant and with NaCl treatment in a form of substrate irrigation (**D**), without rhizobial inoculant and with NaCl treatment in a form of foliage spray (**E**), with rhizobial inoculant and with NaCl treatment in a form of foliage spray (**F**). Each data point represents the mean from four randomly selected containers per treatment with three measurements per container ± SE. * indicates statistically significant differences from respective unburied plants (*p* < 0.05).

**Figure 4:**
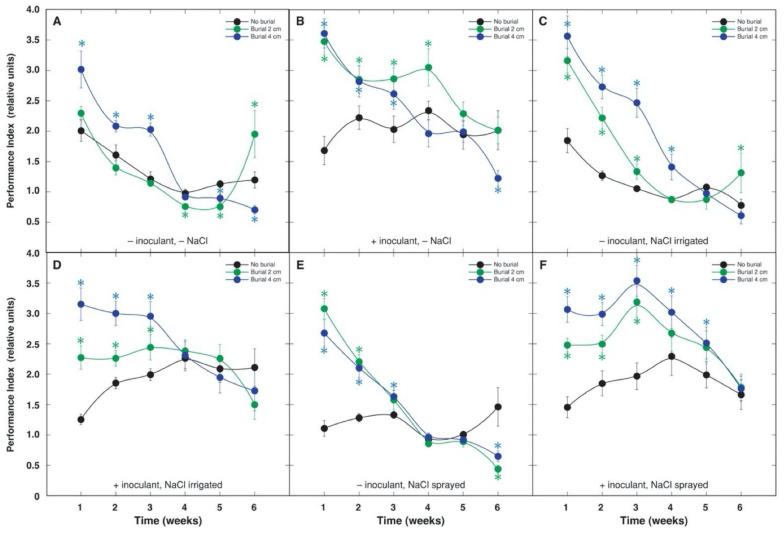
Sand burial effect on time course of chlorophyll *a* fluorescence parameter performance index in leaves of *Anthyllis maritima* without rhizobial inoculant and without NaCl treatment (**A**), with rhizobial inoculant and without NaCl treatment (**B**), without rhizobial inoculant and with NaCl treatment in a form of substrate irrigation (**C**), with rhizobial inoculant and with NaCl treatment in a form of substrate irrigation (**D**), without rhizobial inoculant and with NaCl treatment in a form of foliage spray (**E**), with rhizobial inoculant and with NaCl treatment in a form of foliage spray (**F**). Each data point represents mean from four randomly selected containers per treatment with three measurements per container ± SE. * indicates statistically significant differences from respective unburied plants (*p* < 0.05).

Photosynthesis-related parameters—leaf chlorophyll concentration and chlorophyll *a* fluorescence—were used to monitor the physiological status of *A. maritima* plants throughout the experiment. The most pronounced differences in respect to chlorophyll concentration (Figure 3) and chlorophyll *a* fluorescence parameter performance index (Figure 4) were seen between treatments with and without bacterial inoculant; these values tended to be lower in plants initially grown in sterile substrate without inoculation. The effect was statistically significant starting from the week 4 in the case of chlorophyll concentration (Figure 3) and from the week 3 in the case of performance index (Figure 4), and it was more pronounced later on. When a different intensity of sand burial was compared, leaf chlorophyll concentration increased independently on burial depth, and only in plants not treated with NaCl or irrigated by NaCl; the persistence of the effect was rather short (Figure 3). In contrast, performance index significantly increased by both 2 and 4 cm sand burial in all treatment combinations, except by 2 cm burial in non-inoculated plants without NaCl treatment (Figure 4). The rhizobial inoculation-modified response of performance index to sand burial, resulted in a significant increase in the parameter for plants not treated with NaCl, but in NaCl-treated plants inoculation increased performance index under 2 cm burial.

Results of ANOVA showed that leaf chlorophyll concentration and performance index were highly significantly affected by burial, inoculant and time, but not by NaCl (Table 4). Both parameters were significantly affected by two-way interactions between burial and NaCl, NaCl and time, burial and time, and inoculant and time, but performance index was also significantly affected by interaction between inoculant and NaCl. In addition, performance index was also significantly affected by a three-way interaction between burial, inoculant and time.

### 3.3. Ion Concentration

**Figure 5:**
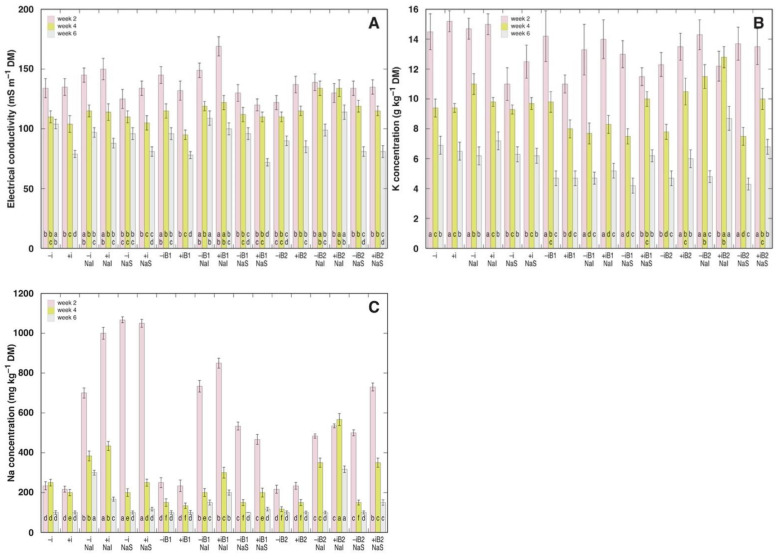
Changes in leaf tissue electrolyte level (measured as electrical conductivity, EC) (**A**), K^+^ concentration (**B**), and Na^+^ concentration (**C**) in leaves of *Anthyllis maritima* plants measured at different points during cultivation as affected by the respective combination of conditions. –i, no rhizobial inoculant; +i, with rhizobial inoculant; NaI, irrigated with 25 mM NaCl; NaS, sprayed with 100 mM NaCl; B1, buried with sand by 2 cm; B2, buried with sand by 4 cm. Data are means from three replicates ± SE. Different letters indicate significant differences (*p* < 0.05) between treatments for the respective time point.

Soluble ion concentrations in leaf water extracts as well as extract electrical conductivity were analyzed three times during the experiment (Figure 5). ANOVA analysis showed that EC was significantly affected by inoculant, NaCl and time (Table 5). Burial, NaCl and time significantly affected K^+^ concentration, but Na^+^ concentration was significantly affected by the same factors as for K^+^ plus by interaction between NaCl and time, and inoculant and time. 

Leaf tissue EC values significantly decreased with time (Figure 5A). Burial had only minor effect on EC, as a certain degree of increase in EC value was evident in some 4 cm burial treatments at later phases of the experiment. NaCl led to increased tissue EC only when substrate irrigation was used as a method of treatment. Inoculation had a negative effect on tissue EC in plants without NaCl buried for 2 cm or without burial, but only at later phases. 

Leaf tissue K^+^ concentration was relatively non-variable between treatments, but it significantly decreased with time (Figure 5B). At two weeks, burial stimulated K^+^ accumulation in NaCl-sprayed plants, but the effect was negative in some other treatment combinations. At the later stages, the consequences of burial on K^+^ concentration were more negative, except for inoculated plants buried with 4 cm of sand and irrigated with NaCl. NaCl treatment led to a significant increase in tissue K^+^ concentration in several treatment combinations, but in some cases significant negative effect was evident. Irrigation as a method of NaCl application was relatively more positive in respect to K^+^ concentration in comparison to application by spray. 

Leaf Na^+^ concentration differed between NaCl-treated and non-treated plants, but Na^+^ concentration was affected by other factors and their combinations, and the concentration significantly decreased with time (Figure 5C). Irrigation with 25 mM NaCl performed both before and after sand burial, led to a more persistent increase of leaf Na^+^ concentration in comparison to spray with 100 mM NaCl. Burial diminished increase in tissue Na^+^ concentration due to NaCl treatment at early stages of the experiment, except in the case of non-inoculated plants irrigated with NaCl. Later on, Na^+^ concentration decreased faster in NaCl-sprayed plants, especially, those without inoculant, but 4 cm sand burial resulted in maintaining high tissue Na^+^ concentration in inoculated plants.

### 3.4. Oxidative Processes and Tissue Damage

**Figure 6:**
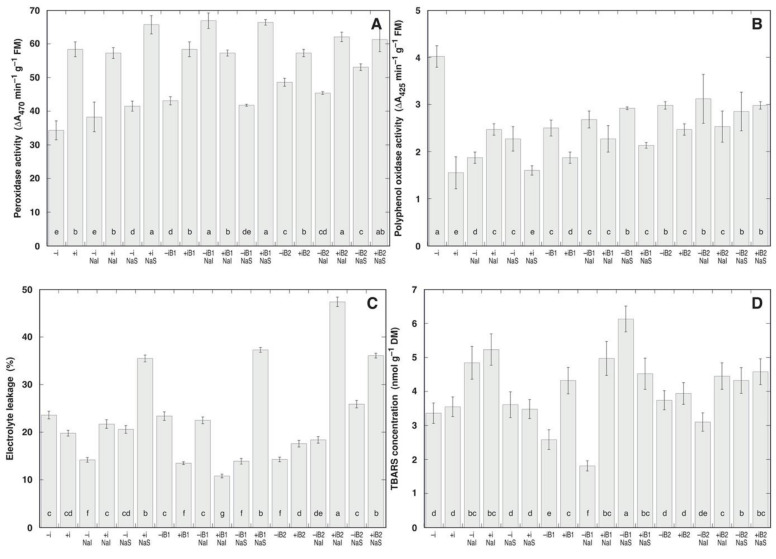
Peroxidase activity (**A**), polyphenol oxidase activity (**B**), electrolyte leakage (**C**), and thiobarbituric acid-reactive substances (TBARS) cocnentration (**D**) in leaves of *Anthyllis maritima* plants after seven weeks of cultivation in the respective combination of conditions. –i, no rhizobial inoculant; +i, with rhizobial inoculant; NaI, irriated with 25 mM NaCl; NaS, sprayed with 100 mM NaCl; B1, buried with sand by 2 cm; B2, buried with sand by 4 cm. Data are means from three replicates ± SE. Different letters indicate significant differences (*p* < 0.05) between treatments.

Peroxidase activity was highly variable between different treatments (Figure 6A) while it was significantly affected only by inoculant (Table 6). Inoculation led to an increase in leaf peroxidase activity in all treatment combinations, except NaCl-irrigated plants buried for 2 cm by sand. Sand burial stimulated peroxidase activity mostly in non-inoculated plants, as inoculation itself led to increased enzyme activity. NaCl treatment increased peroxidase activity in several treatment combinations with no negative impact in the other. 

Similar to peroxidase, polyphenol oxidase activity was significantly affected by inoculant, but to a lesser extent (Figure 6B). This effect was mainly negative, however, for plants with no NaCl treatment buried by 2 cm of sand, inoculation stimulated polyphenol oxidase activity. There was some effect of burial on polyphenol oxidase activity, but it highly depended on other factors. In plants without NaCl treatment, sand burial decreased the activity in non-inoculated plants and increased it in inoculated plants, while in plants irrigated with NaCl, burial increased polyphenol oxidase activity only in non-inoculated plants. In contrast, all treatment combinations of plants sprayed with NaCl showed a significant increase in polyphenol oxidase activity. 

Both the indirect indicator of membrane damage, i.e., rate of electrolyte leakage from tissues (Figure 6C), as well as the biochemical indicator of lipid peroxidation, TBARS concentration (Figure 6D), significantly varied between treatments. There was no tight correlation between the two parameters and none of them were significantly affected by any of the factors, according to the ANOVA analysis (data not shown). However, there was significant effect of experimental factors in several treatment combinations.

## 4. Discussion

### 4.1. Experimental System

To evaluate the effects of sand burial, nitrogen-fixing rhizobial symbiosis and salt treatment, as well as their interactions on *Anthyllis maritima* plants from coastal sand dunes, morphological parameters were used as primary evidence for changes in growth. The physiological status of plants during the experiment was monitored by a non-destructive measurement of leaf chlorophyll concentration and chlorophyll *a* fluorescence analysis. The intensity of oxidative processes as possibly related to defense responses was compared by means of peroxidase and polyphenoloxidase activity analysis; however, possible negative effects were assessed by the measurement of electrolyte leakage from plant tissues and by the measurement of lipid peroxidation indice, concentration of TBARS. 

The experimental system used allowed us to investigate the effects of different single factors, as well as interactions between them on growth and physiological indices of *A. maritima* plants from coastal sand dunes, revealing a cause and effect relationship. The exclusion of rhizobial symbiosis allowed us to significantly modify growth and plant responses to sand burial. However, the effect of inoculation with rhizobia on *A. maritima* plants was controversial. First, physiological performance of plants was significantly improved due to inoculation with bacteria, as indicated by increased photosynthesis-related characteristics, leaf chlorophyll concentration and chlorophyll *a* fluorescence parameter performance index (Figure 3 and Figure 4). Second, plant growth was significantly depressed in plants inoculated with rhizobia. It seems that plants in the present study were not N-limited, as rhizobial inoculation resulted in decreased growth. Similarly, *Medicago sativa* plants fixing N_2_ showed inhibited growth but higher photosynthetic activity when compared with nitrate fed-plants, which was explained as a compensatory mechanism towards carbon cost for rhizobia [32]. 

It seems that active bacterial symbiosis was necessary for maintaining both a high rate of chlorophyll synthesis, as well as photochemical activity of photosynthesis. The results of the present study did not give a clear indication of particular physiological mechanisms of rhizobial inoculation-induced growth inhibition, which was especially pronounced for roots. Still, it is possible to speculate that inoculation of N-supplied plants (adapted to nitrate-ammonium nutrition, as both ammonia-N and nitrate-N were used) resulted in allocating a significant part of photosynthetically fixed carbon to nodules together with the inhibition of symbiotic N fixation [33]. As sand dune plants are well-adapted to N-poor conditions in soil [8], it is possible also that growth inhibition resulted from a feedback control through changes in balance between certain N-containing substances [34]. In further research, it would be possible to exclude the N source on the background of optimum mineral nutrient availability or to use only particular chemical form of N to eliminate any N fertilization-related effect.

The exclusion of symbiotic effects can be performed by using sterile substrate and particular rhizobial inoculants [9]. If no special care has been taken to prevent bacterial contamination by means of watering and other manipulations, the effect can be only short term. In the present study, both sterilized substrate and materials were used in addition to precautions to minimize bacterial contamination during watering. However, when plants grew bigger during the last weeks of the experiment, no special measures were performed to prevent touching between plant shoots. Consequently, at least within for four weeks from the second NaCl treatment symbiotic effects have been excluded.

It needs to be noted that plant response to certain environmental factors (e.g., sand burial) in natural conditions can differ from that in controlled conditions [5,35]. However, in a comparative study with a coastal dune plant *Calystegia soldanella,* it was found that at least biochemical defense machinery was similarly highly active in laboratory-grown plants as in those growing in natural conditions [36].

### 4.2. Effect of Sand Burial

It can be hypothesized that all sand dune plant species have some basic degree of physiological tolerance against burial by sand [5]. Most specialized plants, so-called burial-dependent species, exhibit increased above-ground biomass as a result of burial. These species are important as embryonal dune-formers [37]. 

Physiological mechanism of burial tolerance has been associated mainly with plant ability to emerge from burial, which has been usually interpreted as a burial-induced increase in plant vigor [5]. Characteristics of allocation of biomass between plant parts is a decisive feature for burial susceptibility versus tolerance. Greater shoot growth after burial of the tolerant species could be possible due to resource allocation from roots to shoots, as evident by the decreased biomass of roots [38]. In the present study, *A. maritima*, as a plant from semi-fixed and fixed coastal dunes [16], had only moderate tolerance to burial. First, an increase in shoot biomass was most highly stimulated at 2 cm burial, while petiole height increased at both burial intensities (Table 1). Second, burial did not result in a general reduction of root biomass, as some burial-dependent stimulation of root growth was evident for NaCl-sprayed plants, and growth inhibition was characteristic only for NaCl-irrigated plants. In natural conditions, *A. maritima* is often present together with another endemic species of the Baltic seacoast, *Tragopogon heterospermus* (Asteraceae), which also shows pronounced sand burial tolerance with a characteristic resource allocation from roots to shoots with increased sand accretion intensity [39]. This response was not found for *A. maritima*, in general pointing to the increased photosynthesis rate in buried plants as a mechanism for resource acquisition for growth stimulation. 

An ability to maintain relatively high photosynthetic capacity after burial has been shown to be among important physiological adaptations in sand dune conditions [40]. Thus, partially buried *Cakile edentula* plants had significantly higher leaf chlorophyll concentrations in comparison to control plants [41]. This mechanism is suggested to help plants to compensate for a loss of photosynthetic surface due to burial [40]. Chlorophyll concentration was significantly higher in buried *A. maritima* plants in several treatment combinations, mostly within the first weeks of the experiment (Figure 3). Chlorophyll *a* fluorescence parameter performance index significantly increased in buried plants in all treatment combinations and this effect was relatively more persistent (Figure 4). 

In addition, burial affected the patter of Na^+^ accumulation in plants treated with NaCl: in sand-buried plants, it was initially slower, but an increased level of Na^+^ concentration persisted longer than in non-buried plants (Figure 5C). There is only limited amount of information available on effects of burial on plant mineral nutrition, which mostly concerns increased availability of nutrients for buried plant roots due to freshly deposited sand [42]. In the present study, sand used for burial contained no plant-available nutrients, but K^+^ concentration in leaves of *A. maritima* was significantly affected by burial and this effect was mostly negative (Table 5), especially, for non-inoculated plants 4 to 6 weeks after the treatment (Figure 5B).

### 4.3. Interactions between the Factors

In general, different individual treatments resulted in different effects on *A. maritima* plants when various measured parameters were concerned. This can be explained by more or less specific perception of various environmental factors and corresponding differences in successive metabolic events. At least part of these responses could be related to particular adaptations to respective conditions. It is well known that adaptation of plants to prevailing environmental conditions involves various physiological mechanisms, as in the case of sand accretion and sea water influence for coastal plant species [43,44].

While interaction between sand burial and salinity on the growth of san dune plants can be expected to occur in natural conditions, a very limited amount of information is available on this interaction in controlled conditions. No interaction between sand burial and salt pray on plant growth and reproduction was found in a greenhouse experiment with an annual coastal species *Triplasis purpurea* [15]. Effect of sand burial and salinity as single factors was studied with dune grass species *Leymus arenarius* [43]. However, the effect of partial sand burial on the background of interactions between other factors (soil nutrients and moisture) was studied with *Cakile edentula* in controlled conditions [41]. 

Two types of treatment with NaCl were used in the present study, either to model root zone salinity by substrate irrigation or airborne seawater effect by aerosol spray. As a single factor NaCl treatment had only negligible effect on growth and physiological parameters of *A. maritima*. However, NaCl treatment was important as a factor modulating physiological effect of burial. Thus, NaCl eliminated any negative effect of deep burial (4 cm) on plant growth (e.a., decrease of number of leaves). While increased burial height from 2 to 4 cm led to less vigorous growth of both shoots and roots of *A. maritima* plants, the best adaptation capacity to high burial intensity (4 cm) was evident for plants sprayed with NaCl in the presence of bacterial inoculant. When plants were sprayed with NaCl, even 4 cm burial had positive effect on shoot growth. Inoculation also modified growth response to burial. On the other hand, burial modified shoot growth response to inoculation, as it became more positive in 2 cm buried plants (Table 1). Also, the presence of inoculant dramatically increased the degree of tissue damage, as discussed further, which might be one of the reasons of growth inhibition in rhizobia-treated plants. Moreover, in contrast to sand burial, inoculation in general had no effect on accumulation of K^+^ and Na^+^ in leaves of *A. maritima* (Table 5), as found also in other studies [11,14]. However, Na^+^ concentration in leaves of salt-stressed *Cicer arietinum* plants significantly decreased in *Rhizobium*-inoculated plants [12]. 

Mode of treatment by NaCl had significant effect on NaCl responses, indicating that, in spite of comparable tissue Na^+^ concentrations in plant leaves caused by the two treatments, physiological responses could be different, as found also in the study with *Crambe maritima* [45]. Thus, salt spray usually results in increased leaf thickness and degree of succulence [46].

### 4.4. Physiological Traits and Indicators of Tissue Damage

Physiological and biochemical traits could be important for understanding effect of different factors on plant vigor and growth [14]. Indicators of cellular damage, TBARS (MDA) concentration and electrolyte leakage, are widely used to monitor deleterious biochemical changes imposed by suboptimal conditions at the cellular level. In a well burial-adapted species *Agriophyllum squarrosum* MDA concentration significantly increased only with sand burial at 166% seedling height, but significant increase in tissue electrolyte leakage was evident starting from 75% burial [47]. For *A. maritima*, effect of sand burial on membrane peroxidation depended on both inoculation and NaCl treatment. Paradoxically, no negative effect of burial was evident in plants irrigated with NaCl, as TBARS concentration even significantly decreased in three treatment combinations, but it was strongly negative in all treatment combinations including NaCl spray, as shown by increased TBARS concentration (by 20 to 70%). It seems that intensity of sand burial used the present study, while realistic for coastal dunes of the Baltic Sea, in general was too low and evoked conditions of peroxidative membrane damage only in concert with other factors, as salt spray. 

Sand burial decreased electrolyte leakage in plants without NaCl treatment, but burial effect was rather controversial for NaCl-treated plants, where it strongly depended on the absence/presence of bacterial inoculant. Electrolyte leakage was stimulated by rhizobial inoculation only in buried plants without NaCl treatment and in all plants sprayed with NaCl. High degree of electrolyte leakage (47.4 and 36.1%) was found in plants buried by 4 cm with sand, inoculated with rhizobia and treated with NaCl by irrigation and spray, respectively, but it was also high in other treatment combinations with incoulant and NaCl treatment (Figure 6C). Salinity in a form of 150 mM NaCl treatment increased tissue electrolyte leakage in leaves of legume *Glycine soja* from 10 to 30%, which is roughly comparable with the present results [29]. 

Increase of peroxidase activity in leaves of *A. maritima* plants was a good indicator of rhizobial inoculation (Figure 6A, Table 6). Because of this, sand burial-dependent stimulation of peroxidase activity was seen mostly in non-inoculated plants. So far, increased peroxidase activity has been correlated with cessation of elongation growth [48]. The fact that rhizobial inoculation resulted in decrease of shoot biomass and increase in leaf peroxidase activity is consistent with this idea. In addition, physiological tolerance to sand burial has been associated with efficient induction of enzymatic antioxidant system [49]. In particular, peroxidase activity significantly increased in leaves of *Agriophyllum squarrosum* only starting from sand burial intensity at 133% seedling height [47] If increased peroxidase activity could be a prerequisite to better burial tolerance, then rhizobia-inoculated plants of *A. maritima* should have better shoot growth at more intense sand burial in comparison to non-inoculated plants, but this was not a case in the present study. 

Increase in polyphenol oxidase activity has been associated with responses to wounding (only several plant species) and arthropod herbivores [24]. In addition, cattle grazing induced polyphenol oxidase activity in *Trifolium pratense* plants that was associated with the degree of cellular damage [50]. Inoculation as the only factor with general significant effect on polyphenol oxidase activity in *A. maritima* leaves is consistent with the idea that this enzyme is involved mainly in biotic interactions [51]. Sand burial stimulated polyphenol oxidase activity in all treatment combinations involving rhizobial inoculation, as inoculation itself led to decreased activity of the enzyme. 

Chlorophyll *a* fluorescence parameter total performance index was more sensitive physiological trait in conditions of the present experiment in comparison to leaf chlorophyll concentration. Perofrmance Index appeared to be a good indicator of both rhizobial inoculation as well as sand burial, while leaf chlorophyll concentration was a good indicator of inoculation (Figure 3, Figure 4). Similar dependence of these parameters on rhizobial inoculation were found also in *Trifolium fragiferum* and *Trifolium repens* plants [14].

### 4.5. Importance of the Study for Plant Growth in Natural Conditions

What can be an outcome of the studied interactions in natural conditions in a sandy substrate with low N content but active rhizobial symbiosis? From an ecological point of view, sand burial is considered to be a main factor affecting zonation of plant species in temperate regions, with salt spray playing only a secondary role [6]. It is argued that salt spray episodes mainly occur in late autumn and winter season, when no foliage is present. Similarly, it seems that air-borne salt spray represents only a minor factor for primary and secondary dune plants of the Baltic region in contrast to seashore or salt marsh plants [44]. More stable substrate (semi-fixed and fixed dunes) is located further from sea with less effect from saline water; therefore, in natural conditions, higher burial intensity will be associated with more pronounced salt spray. In this respect, it is interesting that in controlled conditions NaCl had more positive effect for growth of *A. maritima* plants at higher burial intensity showing existence of physiological adaptation(s) to concomitant action of both factors.

In natural conditions *A. maritima* possesses mycorrhizal fungi-associated structures in roots (hyphal coils and vesicles), but the intensity of symbiosis is relatively low [52]. As positive effect of mycorrhizal symbiosis on growth of sand-buried *Agropyron psammophilum* and *Panicum virgatum* plants has been shown [53], presence of active fungal symbiosis in roots of *A. maritima* could indicate complex biotic and abiotic relationships affecting plant responses to sand burial in conditions of coastal dunes. 

In conclusion, strong interaction between sand burial and NaCl treatment was evident in the present study, as NaCl alone had minor effect on *A. maritima* plants, but it significantly affected the effect of burial on growth and physiological indices. Thus, the initial hyopthesis that there will be an interaction between sand burial and salt treatment at the level of physiological responses of *A. maritima* plants was fully confirmed. Moreover, rhizobial symbiosis had significant effect on physiological processes through interaction with both sand burial and NaCl treatment, but the effect was rather controversial: in general, it was positive for photosynthesis-related parameters but negative for growth and tissue integrity indices.

## Figures and Tables

**Table 1:** Morphological characteristics of *Anthyllis maritima* plants after seven weeks of cultivation in the respective combination of conditions.

Treatment	Dry Mass of Shoots (g)	Shoot Water Content (g g^−1^ DM)	Dry Mass of Roots (g)	Root Water Content (g g^−1^ DM)	Leaf Height (cm)	Petiole Height (cm)	Number of Leaves	Number of Shoots
–i	3.72 ± 0.13 cdefg	4.81	0.454 ± 0.084 abc	4.18	7.37 ± 0.23 abc	5.39 ± 0.21 cd	109.6 ± 9.2 a	7.5 ± 0.9 bcde
+i	3.25 ± 0.33 fgh	3.77	0.204 ± 0.041 cd	4.05	6.90 ± 0.24 abcd	5.34 ± 0.25 d	89.8 ± 9.4 bcde	6.5 ± 0.9 e
–iNaI	3.64 ± 0.18 defgh	4.27	0.368 ± 0.115 bcd	5.39	7.13 ± 0.18 abcd	5.45 ± 0.15 cd	102.6 ± 5.8 abc	6.3 ± 0.5 e
+iNaI	3.37 ± 0.26 efgh	4.43	0.178 ± 0.061 cd	5.29	7.34 ± 0.39 abcd	5.72 ± 0.32 bcd	93.8 ± 7.2 abcd	9.8 ± 0.8 abcd
–iNaS	3.32 ± 0.18 efgh	4.69	0.450 ± 0.101 ab	2.87	7.07 ± 0.37 abcd	5.68 ± 0.38 cd	92.8 ± 10.9 abcd	7.8 ± 1.0 bcde
+iNaS	2.65 ± 0.53 h	3.94	0.128 ± 0.032 c	4.08	6.30 ± 0.55 d	5.43 ± 0.43 d	81.6 ± 11.8 cdef	8.5 ± 0.9 abcde
–iB1	4.01 ± 0.45 abcde	4.59	0.448 ± 0.163 abcd	4.69	7.51 ± 0.25 abc	6.00 ± 0.22 abcd	94.2 ± 15.5 ab	7.5 ± 1.0 bcde
+iB1	4.91 ± 0.27 ab	3.81	0.226 ± 0.032 bcd	4.71	7.46 ± 0.14 abc	6.20 ± 0.21 abc	115.6 ± 11.8 a	11.0 ± 1.2 a
–iB1NaI	5.23 ± 0.23 a	4.47	0.216 ± 0.046 c	5.02	7.35 ± 0.26 abc	6.45 ± 0.22 abc	127.8 ± 4.2 a	11.0 ± 0.7 a
+iB1NaI	4.83 ± 0.39 ab	3.91	0.216 ± 0.038 cd	4.88	7.43 ± 0.29 abc	6.03 ± 0.28 abcd	110.8 ± 6.5 a	11.5 ± 0.7 a
–iB1NaS	4.88 ± 0.11 abc	3.96	0.502 ± 0.104 a	4.78	7.41 ± 0.16 abc	6.09 ± 0.13 abcd	109.4 ± 2.9 ab	10.8 ± 1.2 ab
+iB1NaS	3.96 ± 0.62 bcdef	3.98	0.198 ± 0.055 cd	3.09	6.65 ± 0.49 bcd	6.03 ± 0.15 abcd	91.6 ± 15.4 abcd	10.0 ± 1.1 abc
–iB2	4.20 ± 0.34 abcdef	3.86	0.500 ± 0.081 a	4.76	7.91 ± 0.24 ab	6.70 ± 0.20 ab	66.0 ± 3.3 de	7.3 ± 0.8 de
+iB2	2.92 ± 0.63 gh	3.83	0.136 ± 0.040 c	5.49	6.38 ± 0.56 cd	5.73 ± 0.43 cd	58.6 ± 9.3 de	7.3 ± 1.3 de
–iB2NaI	4.03 ± 0.45 absdef	4.16	0.314 ± 0.087 abcd	4.10	7.30 ± 0.33 abc	6.63 ± 0.49 a	75.0 ± 4.4 def	6.3 ± 0.5 e
+iB2NaI	2.57 ± 0.44 h	4.37	0.188 ± 0.044 cd	3.47	7.14 ± 0.77 abcd	6.15 ± 0.23 abc	53.4 ± 8.5 e	5.5 ± 0.9 e
–iB2NaS	4.55 ± 0.20 abcd	3.88	0.540 ± 0.169 abc	6.83	7.73 ± 0.34 ab	6.63 ± 0.25 ab	82.6 ± 8.0 cdef	9.0 ± 2.0 bcde
+iB2NaS	4.16 ± 0.57 abcde	4.10	0.296 ± 0.045 abcd	2.95	7.76 ± 0.86 a	6.10 ± 0.34 abc	82.2 ± 8.4 bcde	8.0 ± 0.8 bcde

–i, no rhizobial inoculant; +i, with rhizobial inoculant; NaI, irrigated with 25 mM NaCl; NaS, sprayed with 100 mM NaCl; B1, buried with sand by 2 cm; B2, buried with sand by 4 cm. Data are means from six replicates ± SE. Different letters indicate significant differences (*p* < 0.05) between treatments for the respective parameter.

**Table 2:** ANOVA analysis of shoot and root mass parameters.

Source of Variation	df	Shoot FM		Shoot DM		Root FM		Root DM	
		Mean Square	*F*	Mean Square	*F*	Mean Square	*F*	Mean Square	*F*
Burial	2	302.9	9.57 ***	13.44	18.1 ***	0.28	0.26	0.006	0.17
Inoculant	1	344.5	10.88 **	6.81	9.17 **	37.39	34.98 ***	1.091	32.86 ***
NaCl	2	10.5	0.72	0.10	0.14	1.34	1.25	0.084	2.52
Burial × inoculant	2	8.1	0.26	1.57	2.12	0.24	0.23	0.012	0.37
Burial × NaCl	4	62.5	1.97	2.46	3.31 *	1.44	1.35	0.017	0.50
Inoculant × NaCl	2	0.8	0.03	0.40	0.55	2.16	2.02	0.074	2.24
Burial × inoculant × NaCl	4	38.0	1.20	1.35	1.82	0.80	0.74	0.018	0.53
Residuals	72	31.7		0.74		1.07		0.033	

* *p* < 0.05, ** *p* < 0.01, *** *p* < 0.001.

**Table 3:** ANOVA analysis of shoot morphological parameters.

Source of Variation	df	Leaf Height		Petiole Height		Number of Leaves		Number of Shoots	
		Mean Square	*F*	Mean Square	*F*	Mean Square	*F*	Mean Square	*F*
Burial	2	1.040	1.19	5.545	13.19 ***	11,547	27.27 ***	84.31	24.40 ***
Inoculant	1	3.238	3.70	1.449	3.45	1895	4.48 *	7.51	2.17
NaCl	2	0.143	0.16	0.237	0.56	202	0.48	10.81	3.13 *
Burial × inoculant	2	0.183	0.21	0.930	2.21	147	0.35	6.98	2.02
Burial × NaCl	4	1.192	1.36	0.040	0.10	1231	2.91 *	10.13	2.93 *
Inoculant × NaCl	2	1.073	1.23	0.010	0.02	363	0.86	4.88	1.41
Burial × inoculant × NaCl	4	0.940	1.07	0.289	0.69	634	1.50	9.79	2.83 *
Residuals	72	0.875		0.420		423		3.46	

* *p* < 0.05, *** *p* < 0.001.

**Table 4:** ANOVA analysis of chlorophyll concentration and performance index leaves of *Anthyllis maritima* plants.

Source of Variation	df	Chlorophyll Concentration		Performance Index	
		Mean Square	*F*	Mean Square	*F*
Burial	2	72,168	28.84 ***	30.01	62.43 ***
Inoculant	1	507,142	202.64 ***	205.60	427.65 ***
NaCl	2	5227	2.09	0.21	0.43
Burial × inoculant	5	361,662	144.51 ***	37.15	77.27 ***
Burial × NaCl	2	5003	2.00	3.38	7.03 ***
Inoculant × NaCl	4	11,913	4.76 ***	2.00	4.16 **
Burial × inoculant × NaCl	2	1040	0.42	5.20	10.82 ***
Residuals	10	9934	3.97 ***	1.04	2.15 *

* *p* < 0.05, ** *p* < 0.01., *** *p* < 0.001.

**Table 5:** ANOVA analysis of tissue electrical conductivity and concentration of K^+^ and Na^+^ in leaves of *Anthyllis maritima* plants.

Source of Variation	df	EC		K^+^ Concentration		Na^+^ Concentration	
		Mean Square	*F*	Mean Square	*F*	Mean Square	*F*
Burial	2	0.022	1.22	2915	5.63 **	23.2	4.61 *
Inoculant	1	0.102	5.65 *	1753	3.38	15.9	3.16
NaCl	2	0.427	23.55 ***	2047	3.95 *	134.2	26.6 ***
Time	2	3.559	196.43 ***	101 636	195.92 ***	338.0	67.12 ***
Burial × Inoculant	2	0.034	1.90	1068	2.06	4.9	0.98
Burial × NaCl	4	0.030	1.63	615	1.19	5.3	1.06
Inoculant × NaCl	2	0.040	2.21	462	0.89	5.8	1.15
NaCl × Time	4	0.010	0.58	270	0.52	47.4	9.41 ***
Burial × Time	4	0.052	2.89 *	240	0.46	14.1	2.81 *
Inoculant × Time	2	0.078	4.33 *	1064	2.05	1.2	0.25
Burial × Inoculant × NaCl	4	0.026	1.45	497	0.96	0.8	0.17
Residuals	24	0.018		519		5.0	

* *p* < 0.05, ** *p* < 0.01, *** *p* < 0.001.

**Table 6:** ANOVA analysis of peroxidase and polyphenol oxidase activity in leaves of *Anthyllis maritima* plants.

Source of Variation	df	Peroxidase Activity		Polyphenol Oxidase Activity	
		Mean Square	*F*	Mean Square	*F*
Burial	2	71.1	3.75	0.763	2.50
Inoculant	1	1599.7	84.45 ***	1.923	6.30 *
NaCl	2	19.1	1.01	0.099	0.32
Residuals	12	18.9		0.305	

* *p* < 0.05, *** *p* < 0.001.

## Data Availability

All data reported here is available from the authors upon request.
